# mHealth Impact on Gait and Dynamic Balance Outcomes in Neurorehabilitation: Systematic Review and Meta-analysis

**DOI:** 10.1007/s10916-023-01963-y

**Published:** 2023-07-18

**Authors:** Marta Moreno-Ligero, David Lucena-Anton, Alejandro Salazar, Inmaculada Failde, Jose A. Moral-Munoz

**Affiliations:** 1 Department of Biomedicine, Biotechnology and Public Health, Faculty of Nursing and Physiotherapy, University of Cadiz, 11009 Cadiz, Spain; 2 Department of Nursing and Physiotherapy, Faculty of Nursing and Physiotherapy, University of Cadiz, 11009 Cadiz, Spain; 3 Biomedical Research and Innovation Institute of Cadiz (INiBICA), University of Cadiz, 11009 Cadiz, Spain; 4 Department of Statistics and Operational Research, School of Engineering, University of Cadiz, 11510 Puerto Real, Cadiz, Spain

**Keywords:** mHealth, Mobile applications, Gait, Balance, Rehabilitation, Neurological disorders

## Abstract

Gait and dynamic balance are two main goals in neurorehabilitation that mHealth systems could address. To analyze the impact of using mHealth systems on gait and dynamic balance outcomes in subjects with neurological disorders. Randomized controlled trials (RCT) published in PubMed, Web of Science, Scopus, and PEDro databases were searched up to April 2023. Studies including adults with neurological disorders, analyzing the effectiveness of mHealth systems on gait and dynamic balance compared with conventional therapy and/or not intervention, were included. The PEDro scale and the Cochrane Collaboration’s 2.0 tool were used for the methodological quality and risk of bias assessment. The Review Manager 5.4 software was used to obtain meta-analyses. 13 RCT were included in the systematic review and 11 in the meta-analyses, involving 528 subjects. A total of 21 mobile applications were identified for gait and balance training, and to enhance physical activity behaviors. There were significant differences in gait parameters, speed by 0.10 s/m (95% confidence interval (CI)=0.07,0.13;p<0.001), cadence by 8.01 steps/min (95%CI=3.30,12.72;p<0.001), affected step length by 8.89 cm (95%CI=4.88,12.90;p<0.001), non-affected step length by 8.08 cm (5%CI=2.64,13.51;p=0.004), and in dynamic balance, Timed Up and Go by -7.15 s (95%CI=-9.30,-4.99;p<0.001), and mobility subscale of Posture Assessment Scale for Stroke by 1.71 points (95%CI=1.38,2.04;p<0.001). Our findings suggested the use of mHealth systems for improving gait in subjects with neurological disorders, but controversial results on dynamics balance recovery were obtained. However, the quality of evidence is insufficient to strongly recommend them, so further research is needed.

**Supplementary Information:**

The online version contains supplementary material available at 10.1007/s10916-023-01963-y.

## Introduction

Motor impairments represent a leading cause for the limitation of functional activities and disability in subjects with neurological disorders [1–4]. In particular, they require gait and balance for community ambulation and autonomy, which are indirectly related to participation, one domain in the International Classification of Functioning, Disability and Health (ICF) model [5, 6]. Moreover, a loss of postural control during gait increases the likelihood of falls [7, 8]. Therefore, a key goal of neurorehabilitation is to restore mobility through gait [9, 10].

In that way, mHealth, mobile device-based healthcare [11], involves the practice of medicine and public health based on mobile devices to improve and promote health status [12]. It is considered a domain of telerehabilitation when it applies to rehabilitation programs [13]. The interest in mHealth has particularly boomed because of the growth of smartphone access and the use of mobile applications, commonly called “*apps*” [14]. In the neurorehabilitation field, there are apps to monitor vital signs [15], detect neurological symptomatology [16–18], track neurodegenerative diseases [19–21], treat motor and cognitive impairments [22–25], among others. In that sense, Dorsey et al. [26] reviewed the global proliferation and current state of telerehabilitation in neurological disorders. They highlighted the potential to increase access to neurological care and education by telerehabilitation. Likewise, Dobkin [27] suggested mHealth enables therapists to remotely monitor adherence and to obtain continuous outcomes measurements over time, allowing the assessment of goal attainment in the daily routine. Consequently, the use of these technologies facilitates the self-management of the rehabilitation process and becomes the subject more active, increasing participation. Because of this background, mHealth could complement multimodal programs in neurorehabilitation, making it more effective, including different sensorial feedback, motor experiences, and increasing the subject’s motivation [28].

Concerning the current scientific literature, some reviews are analyzing the potential use of apps in subjects with neurological disorders [13, 29–31]. Nevertheless, authors mainly analyzed commercial apps available in repositories, whose scientific evidence was slightly based on randomized clinical trials (RCT). To our knowledge, there is not a review and meta-analysis examining the use of mHealth systems in neurorehabilitation, specifically focused on their impact on gait and balance outcomes. Consequently, the main aim of this study was to analyze the impact of the use of mHealth systems on gait and dynamic balance outcomes in subjects with neurological disorders, performing a systematic review and meta-analysis.

## Methods

### Search strategy

The Preferred Reporting Items for Systematic Reviews and Meta-Analyses (PRISMA) guidelines for systematic reviews of randomized controlled trials [32] was followed to report the present systematic review and meta-analysis (Online resource 1). The search was performed up to April 2023 in the following databases: PubMed, Web of Science, Scopus, and Physiotherapy Evidence Database (PEDro). It was adapted for the rest of the databases from an initial search of the PubMed database (Online resource 2). No filters were used in terms of language or publication date.

### Eligibility criteria

The inclusion criteria were defined according to the PICOS framework [33] (Population, Intervention, Comparison, Outcomes, Studies): i) RCT including adults (over 18 years old) with neurological disorders, ii) comparing mHealth systems (smartphones or tablets) with a control group, iii) mHealth systems used during gait and balance training, apps based on physical activity (PA) programs in real-world settings, and mobile apps providing feedback to enhance self-patient management and participation to rehabilitation programs, iv) Control group was based on conventional therapy (physical and/or occupational therapies, such as specific gait and balance training, home-based PA programs and active behavior recommendations) and/or not intervention, v) gait and dynamic balance as outcomes.

The exclusion criteria were: i) Systems based on videoconferencing platforms (e.g. Skype, Zoom, etc.), virtual reality glasses, or websites, ii) Studies related to cost-effectiveness and usability of mHealth systems.

### Study selection

Two authors (M.M.L and J.A.M.M.) retrieved and screened the studies by the titles and abstracts independently, removing duplicate articles and identifying potentially relevant articles according to the pre-established criteria. Subsequently, full texts of the retrieved records were downloaded and analyzed for final inclusion in the systematic review. An additional reviewer (D.L.A.) was considered for consensus when needed. Some information was extracted to detail the studies characteristics: authors, year of publication, country, characteristics of the participants (number of participants in both groups, mean age, neurological disorder), and the intervention characteristics (types, session length and frequency, total duration, outcomes measured, measurement instruments, and results). In addition, each mHealth system employed as an intervention was identified, specifying its purpose and main features.

### Quality assessment

The PEDro scale [34] was used to evaluate the methodological quality of the studies. A study scored as 6 or more is considered having good methodological quality (6-8: good; 9-10: excellent) and scored as 5 or less is considered being of acceptable or poor quality (4-5: acceptable; <4: poor) [35]. The Cochrane Risk of Bias (RoB) Collaboration’s 2.0 tool [36] was used for assessing the risk of bias. This evaluation covers five domains of bias: (1) bias arising from the randomization process, (2) bias due to deviations from intended interventions, (3) bias due to missing outcome data, (4) bias in measurement of the outcome, and (5) bias in selection of the reported result. The RoB of each domain was categorized as: “low risk”, “some concerns”, and “high risk”. Also, an overall RoB assessment of the RCT was performed according to the recommendations in the guidance document. Those assessments were performed by two authors (M.M.L and J.A.M.M.) independently. When there was a difference in the scores between the authors, the final decision was discussed with a third author (D.L.A.).

### Statistical analysis

Several meta-analyses were performed with The Review Manager 5.4 software to summarize the comparisons of the use of mHealth systems (experimental group) versus a control group. The studies were classified based on the measuring instrument to assess gait or dynamic balance. The effect measure was the mean difference (MD), reported along with their 95% confidence intervals (CI). The instructions of the Cochrane Handbook for Systematic Reviews of Interventions for obtaining standard deviation from confidence intervals and change-from-baseline standard deviation were employed when needed. After checking for homogeneity with the chi-square test and the I^2^ statistic (0-40% might not be important; 30-60% may represent moderate heterogeneity; 50-90% may represent substantial heterogeneity; and 75-100% considerable heterogeneity) [37], a fixed-effect model was used when no heterogeneity was detected, and a random model was used otherwise. The significance level was set at alpha=0.05, and the results were presented in forest plots.

## Results

**Fig. 1:**
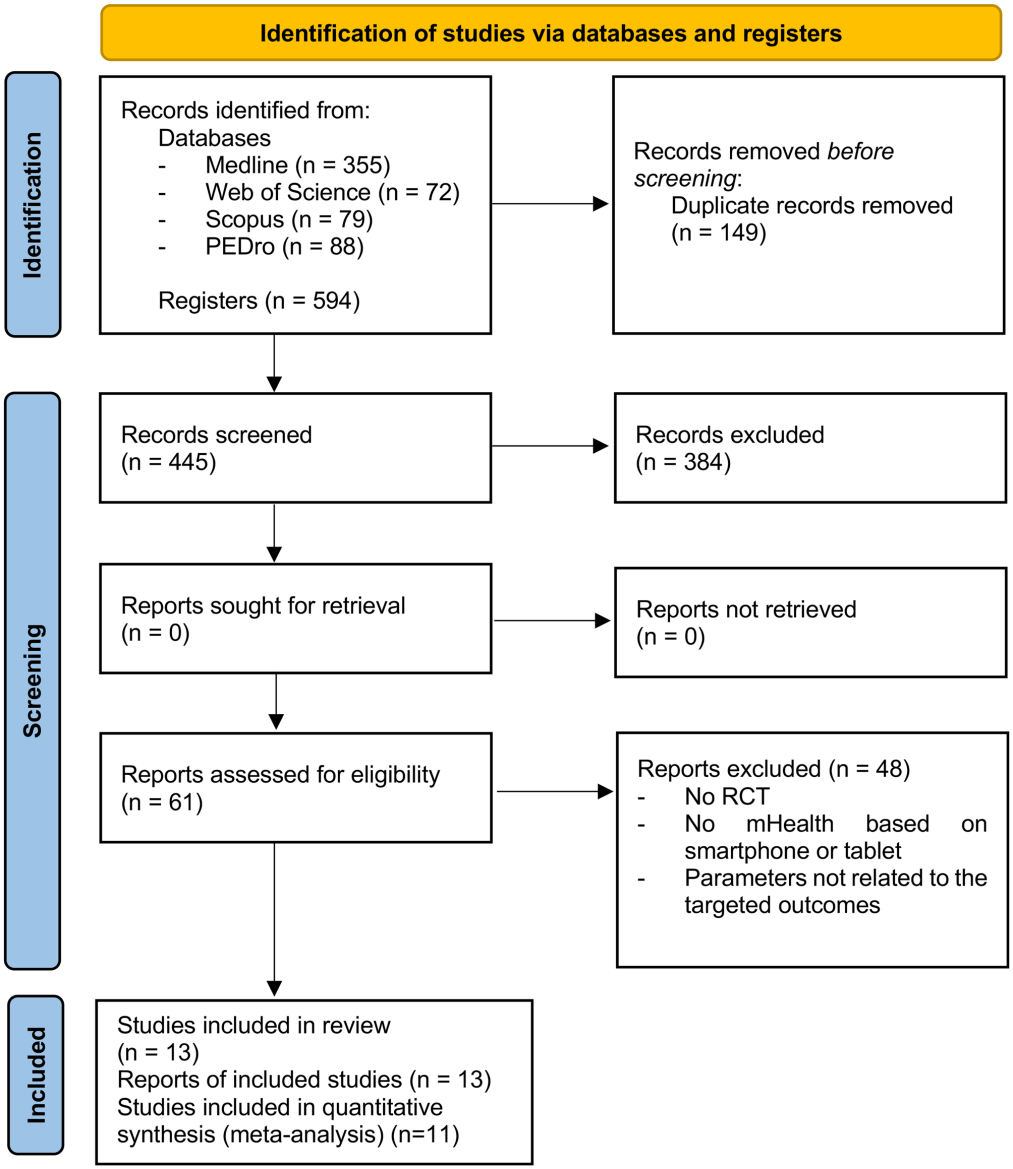
Information flow diagram of the different phases of the systematic review.

A total of 594 articles were retrieved during the initial search and 13 RCT were included in the systematic review and 11 in the meta-analysis (Fig. 1). The studies by Asano et al. [38] and Hankinson et al. [39] were excluded because it did not provide the necessary data for the meta-analysis. In that way, 528 subjects participated in the included studies.

### Studies characteristics

The average age was 60.4, although three documents did not provide information [39–41]. All studies included both genders and the most common disease was stroke, studied in eight articles [38, 42–47] (Table 1).

**Table 1:** Participants and interventions characteristics

**STUDY** (Authors, year, and country)	**NEUROLOGICAL CONDITION**	**GROUPS**	**AGES**	**TYPE OF INTERVENTION**		**TREATMENT PROTOCOL** (Session duration and frequency, and total duration*)*		**OUTCOME** **MEASURES**	**MEASURING INSTRUMENTS**	**RESULTS**
				**mHealth Group**	**Control Group**	**mHealth Group**	**Control Group**			
Kim et al., 2012 [47] South Korea	Stroke	N = 18 mHealth G: 9 Control G: 9	mHealth G: 53.311.8 Control G: 51.813.7	Gait training based on rhythmic auditory stimulation by an app + CGI	Physical therapy	-Session: 30 min of mHealth per day + 30 min of CGI twice a day -Frequency: 3 sessions (mHealth) and 5 sessions (CGI) per week -Total duration: 5 weeks	-Session: 30 min twice a day -Frequency: 5 sessions per week -Total duration: 5 weeks.	-Spatiotemporal gait parameters -Balance and postural stability -Fall risk	GAITRite system; FSST; TUG; DGI; FAC; ABC Scale	Spatiotemporal gait outcomes improved in both groups. In mHealth group, the difference pre-post intervention in speed, cadence and stride length were significant (*p*<0.05). By the inter-group analysis, the mHealth group showed significant improvements in scores on the ABC scale, TUG, DGI (*p*<0.001), compared with the control group.
Shin and Song, 2016 [45] South Korea	Stroke	N = 24 mHealth G: 12 Control G: 12	mHealth G: 57.75 ± 14.03 Control G: 59.259.75	Balance training by an app + CGI	Physical and occupational therapies, and electrical stimulation therapy	-Session: 80 min of CGI + 20 min of mHealth per day -Frequency: 3 sessions (mHealth) and 5 sessions (CGI) per week -Total duration: 4 weeks	-Session: 80 min per day -Frequency: 5 sessions per week -Total duration: 4 weeks.	-Dynamic and static balance	Good Balance system; mRFT; TUG; TIS.	Improvements of static and dynamic balance (*p*<0.05) on sitting, TUG test (*p*<0.001) and TIS score (*p*=0.02) were significantly higher in mHealth group compared with control group after the 4-weeks. Regarding changes in trunk performance (mRFT), a significantly increased was observed in both groups.
Lee et al., 2017 [44] South Korea	Stroke	N = 24 mHealth G: 12 Control G: 12	mHealth G: 55.11±14.62 Control G: 52.065.54	Speed-Interactive Treadmill Training (SITT) based on app	Conventional SITT	-Session: 35 min per day -Frequency: 3 sessions per week -Total duration: 6 weeks	-Session: 35 min per day -Frequency: 3 sessions per week -Total duration: 6 weeks	-Spatiotemporal gait parameters and gait symmetry	OptoGait system	An overall improvement in spatiotemporal gait parameters was observed in both groups. However, significant changes between mHealth and control groups were about stride length (*p*=0.042) and affected and non-affected step (*p*=0.29). According to gait symmetry, both analysis (intra and intergroup) did not show significant improvement.
Grau-Pellicer et al., 2019 [46] Spain	Stroke	N = 41 mHealth G: 24 Control G: 17	mHealth G: 62.96±11.87 Control G: 68.5311.53	Monitoring PA parameters and health-related outcomes by the apps + Home-based PA program	Physical and occupational therapies	-Session: 1 hour of PA per day + daily PA monitoring -Frequency: 2 sessions per week -Total duration: 8 weeks	-Session: 1 hour of PA per day -Frequency: 2 sessions per week -Total duration: 12 weeks	-PA and gait performance -Balance and postural stability	PA and sedentary daily time; 10MWT; 6MWT; TUG.	Changes in community ambulation and sitting time per day were statistical significantly in mHealth group (*p*<0.05). Both groups significantly improved the gait speed (*p*<0.05), however gait performance (6MWT) increased only in mHealth group. According to dynamic balance and the risk of falls, the TUG time decreased significantly by 3.46 seconds in mHealth group. This value is below the 14 s cutoff value (9.59±3.15), considering as “non-fallers”.
Asano et al., 2021 [38] Singapore	Stroke	N = 124 mHealth G: 61 Control G: 63	mHealth G: 63.8 Control G: 64.4	Prescribed home-based PA program and video-feedback provided by an app + CGI (if participants wished)	Physiotherapy and occupational therapies	-Session: 1 hour per day -Frequency: 5 sessions (mHealth) and 1-2 sessions (CGI) per week -Total duration: 12weeks	-Session: 1 hour per day -Frequency: 1-2 sessions per week -Total duration: 12 weeks	-Gait speed and endurance	5MWT; 2MWT	At 12 weeks post-intervention, both groups showed improvements in gait speed measured by the 5MWT (*p*=0.026) and gait endurance by the 2MWT (*p*=0.034) from baseline. However, there was no difference between groups.
Ginis et al. 2016 [41] Belgium and Israel	Parkinson Disease	N = 40 mHealth G: 22 Control G: 18	ND	Gait training by apps	Conventional gait training	-Session: 30 min (ABF-gait app) per day + 30 min (FOG-cue app) per day only for participant with FOG -Frequency: 3 sessions per week -Total duration: 6 weeks	-Session: 30 min per day -Frequency: 3 sessions per week -Total duration: 6 weeks	-Spatiotemporal gait parameters -Balance and physical condition	Protokinetics instrumented walkway; FSST; MiniBESTest; FES-I; PASE; 2MWT.	Improvements in speed gait and stride length after training period were observed in both groups (*p*<0.001), but there were not significant differences between them (*p*>0.05). Significant improvements in dynamic balance by the FSST (*p*<0.05) were achieved by the mHealth group, but there was not a significant interaction effect of time by groups (*p*=0.09).
Ellis et al., 2018 [48] United States of America	Parkinson Disease	N = 51 mHealth G: 26 Control G: 25	mHealth G: 64.8±8.5 Control G: 63.3±10.6	App-based PA program + passive monitoring	Paper-based PA intervention + passive monitoring	-Session: 5-7 active exercises for 3 day/week + Daily PA monitoring. -Frequency: 3 sessions per week -Total duration: 12 weeks.	-Session: 5-7 active exercises for 3 day/week + Daily PA monitoring -Frequency: 3 sessions per week -Total duration: 12 weeks.	-PA level and gait performance	StepWatch Activity Monitor device; 6MWT.	Significant results (*p*=0.02) and clinical changes were observed in mHealth group for the 6MWT. However, there were not significant differences between groups. Also, both groups improved daily walking minutes and distance walked per day from the beginning, but the difference between groups was not statistically significance.
Nasseri et al., 2020 [50] Germany	Multiple sclerosis	N = 38 mHealth G: 18 Control G: 20	mHealth G: 49.6±8.5 Control G: 52.5±7.3	Evidence-based patient information (EBPI) by an app, including activity feedback, texts, figures, and videos.	General information about effects of exercising based on a leaflet	-Session duration not defined -Frequency: Weekly access to app content. -Total duration: 12 weeks	-Session duration and frequency were not defined -Total duration: 12 weeks	-Gait endurance and performance	6MWT; 2MWT; T25FW; Timed Tandem Walk	Despite both groups improve outcome measures in 6MWT and 2MWT, there were not statistically significant differences from baseline to follow-up for any test (*p*=0.22 and *p*=0.27, respectively). As well, the difference between groups were no significant in both 6MWT (*p*=0.45) and 2MWT (*p*=0.64).
Plow and Golding, 2017 [49] United States of America	Neurological conditions	N = 46 mHealth G: 15 Control G: 15 G3: 16	*(Average age)* 57.89.48	Self-management and app-based PA program + Phone calls Group 3: Paper-based PA program + Phone calls	Generic education on PA and health information and behaviors + Phone calls	-Session: 30 min of PA per day + daily PA self-management -Frequency: 3-5 sessions of PA per week + 3 follow-up phone calls -Total duration: 7 weeks	Similar length of time as phone calls Frequency: 3 follow-up phone calls -Total duration: 7 weeks	-Physical and walking performance -PA levels and behaviors	6MWT; 1min chair stands; 1min arm curls. PADs; PROMIS	There is non-statistically significant difference in 6MWT between mHealth and control group (*p*=0.16). Regarding PA activity and behaviors, participants in mHealth group increased their engagement in PA (*p*<0.05) while in control group decreased it.
Li et al., 2020 [40] Australia	Neurological conditions	N = 38 mHealth G: 22 Control G: 16	ND	App-based PA program + CGI	Physical therapy	-Session: not defined -Intervention: depends on the length of patients stay in rehabilitation	Session: not defined Intervention: depends on the length of patients stay in rehabilitation	-Total supplementary PA dosage (mHealth G) -Gait endurance, gait speed, dynamic balance, and level of disability	Frequency of app use and repetitions of exercise performed 6MWT;10MWT; TUG; FIM.	Both groups demonstrated significant changes for all functional outcome measures (*p*<0.01). However, there were not significant differences in 6MWT (*p*=0.5), 10MWT (*p*=0.2) and TUG (*p*=0.5) between groups.
Hankinson et al., 2022 [39] Australia	Stroke	N=22 mHealth G: 10 Control G: 12	ND	Music-motor training app	Usual care stroke rehabilitation	-Session: 20-min music-motor therapy -Frequency: 3 sessions per week (Monday, Wednesday, and Friday) -Total duration: 6 weeks	-Sessions and frequency not defined	-Upper and lower limb motor function -Adherence	Fugl-Meyer Assessment (FMA) of Motor Recovery score	Upper limb score improved in both intervention and control groups at 3-week follow-up.
Salgueiro et al., 2022 [43] Spain	Stroke	N = 30 mHealth G: 15 Control G: 15	mHealth G: 57.27±14.35 Control G: 64.53±9.4	Home-based core-stability based on a telerehabilitation app + CGI	Conventional physiotherapy (muscle stretching, passive and functional mobilization, sitting and standing posture and gait, task and aerobic training)	-Session: depend on the number of exercises and time spent in performing them. -Frequency: 5 days per week -Total duration: 12 weeks	-Session: one-hour face-to-face -Frequency: 2 times per week -Total duration: 12 weeks	-Trunk performance and sitting balance -Standing balance -Adherence	S-TIS2.0; S-FIST; S-PASS, BBS; number of falls registered; G-Walk accelerometer system.	Regarding trunk function measures, significant improvements in sitting balance by the S-TIS 2.0 were observed in both groups, but the mHealth group scored superior to control in balance-scale and total score. Also, for coordination-scale, only mHealth group achieved statistically significant difference post-intervention. In relation to the standing balance, significant differences in the S-PASS mobility section were observed in mHealth group pre-post intervention, but not when compared with the control group. Also, neither in the S-PASS balance section nor S-PASS total score were observed statistically significant differences in intergroup analysis. Regarding the BBS, statistical significance of the increasing score were observed in intragroup analysis for both mHealth and control groups, but did not achieved when compared between groups.
Aphiphaksakul and Siriphorn, 2022 [42] Indonesia	Stroke	N = 32 mHealth G: 16 Control G: 16	mHealth G: 59.38±11.09 Control G: 59.38±10.80	Home-based exercise utilizing a balance disc with input from a smartphone inclinometer app + CGI	Conventional home rehabilitation program	-Session: 30-minute sitting balance training program -Frequency: 5 days per week -Total duration: 4 weeks	-Session: 60 minutes -Frequency: 5 days per week -Total duration: 4 weeks	-Sitting balance performance -Daily life activities	PASS; FIST; BI.	Patients in both groups significantly improved their FIST, PASS total score and sub-scales, and BI at post-test. Intergroup analysis showed that the mHealth group scored significantly higher on the PASS changing posture sub-scales (*p*=0.010) and BI (*p*=0.018). In fact, the BI group difference was clinically significant. No statistically significant difference was observed between groups in any other measurements.

*ABC scale* Activities-Specific Balance Confidence Scale, *ABF*, Audio-biofeedback, *App* application, *CGI* Control group intervention, *DGI* Dynamic Gait Index, *FAC* Functional Ambulation Classification, *FES-I* Falls Efficacy Scale-International, *FIM* Functional Independence Measure, *FOG* Freezing of Gait, *FSST* Four Step Square Test, *G* group, *Min* minutes, *MiniBESTest* Balance Evaluation System Test (shorten form), *mRFT* Modified Relay Feedback Test, *ND* no data, *PA* Physical activity, *PADs* Physical Activity and Disability Survey, *PASE* Physical Activity Scale for the Elderly, *TIS* Trunk Impairment Scale, *TUG* Time Up & Go, *T25FW* Timed 25 Foot Walk, *2MWT* 2-Minute Walk Test, *6MWT* 6-Minute Walk Test, *10MWT* 10-Meter Walk Test

Regarding the mHealth systems, they can be classified as those specifically focused on gait training, balance training or those based on home-based PA programs. First, gait training was performed by four different apps: i) ZyMiMetronome App [47] and GotRhythm App [39], two metronome apps that provided a rhythmic auditory stimulation during gait training; ii) Virtual Active App [44], used for speed-interactive treadmill training providing visual feedback through filmed world-famous landscapes; and iii) Audio-biofeedback (ABF)-gait and Freezing of Gait (FOG)-cue Apps [41], part of the CuPiD system, provided biofeedback by verbal instructions and visual information, respectively, during gait recovery. Second, for balance training, a group of apps called CSMi [45] was used, included in the SPVFTCT (smartphone-based visual feedback trunk control training) system, designed for trunk control training. It allowed real-time feedback through the monitoring of weight shifts during the execution of the proposed goal task. Similarly, the Compass Inclinometer App [42] was used for providing patients visual feedback while performing a home-based sitting balance training program. Another mHealth system used for balance training was the Farmalarm App [43], which guided home-based core stability exercise (CSE) through description, photo, and video of each exercise, and recorded its performance. Finally, we found apps providing exercise programs to enhance PA. They were not specifically designed for training gait and balance, but the impact of these apps on these outcomes was assessed. The apps used were Wellpepper App [48], Fitlab Training App [46], Lose it! [49], iPro Habit Tracker Apps [49], Memories App [49], Pt Pal App [40], Patient Information App (PIA) [50], and ad-hoc app [38]. They provided home-based PA programs including videos and prescription information, allowing the monitoring (behaviors, symptoms, or goal achievement), the guidance of the PA performance (visual and/or auditory), and the communication with their physical therapist. More specifications about the mHealth systems employed are shown in Table 2.

**Table 2:** mHealth systems features

**NEUROLOGICAL CONDITION**	**MHEALTH SYSTEM NAME**	**PURPOSE**	**MAINLY APP FEATURES**
Stroke	ZyMi Metronome App [47]	Train gait by rhythmic auditory stimulation (RAS).	It is a metronome app which provide a rhythmic auditory stimulation trough the subject’s earphone.
	CSMi Apps [45]	Train balance by visual and auditory feedback.	There was a group of apps which were designed to allow real-time visual auditory feedback information during the balance training goal tasks. The apps and their main purpose are: (1) The CSMi Center of Pressure (to measure the changes in the subject’s center of pressure during sitting posture); (2) The CSMi Limits of Stability (to measure the subject’s capacity to stabilize their balance); (3) The CSMi Weight Bearing Front-Back and CSMi Weight Bearing Left-Right (both apps show the subject’s front-back and right-left weight position); (4) The CSMi Weight Shift (to measure the subject’s ability to change their pressure center from different directions); (5) The CSMi Animal Adventure (it is a game that measures the ability of the subject to regulate the balance at differing locations around the neutral position).
	Virtual Active App [44]	Train gait with speed-interactive treadmill based on visual feedback.	The app displays filmed images of world-famous landscapes (mountains, valleys, and cities) while subjects are walking on a speed-treadmill. The app controls the speed of the video according to the subject’s gait speed.
	Fitlab Training and Fitlab Test Apps [46]	Monitor physical activity parameters and health-related outcomes.	The two apps were used: (1) to record physical activity adherence and the walking distance and walking speed; (2) to measure mood states, effort, recovery, fatigue, and well-being by questionnaires; (3) to provide bidirectional feedback (subjects can see their progress and exchange text-messages with the research staff).
	Ad-hoc App [38]	Prescribe a home-based physical activity program.	The app provides the exercises prescribed by video clips with the instructions to perform each exercise, allowing subjects to familiarize with the exercises. The sensors record and monitor the number of repetitions performed, and the app provides corrective visual and audio feedback during exercises execution. It also allows to self-record the exercises performed for further review.
	Farmalarm App [43]	Home-based core-stability exercises (CSE) program.	The app guides a CSE program through description, photo, and video of a total of 32 exercises. Also, the app records the performance of the exercises proposed. The introduction of the exercises was based on the difficult of the position of the exercises, from the supine to sitting on an unstable base.
	GoRhythm App [39]	Individualized music-motor therapy.	The app includes personal music, wireless wearables sensors and real-time auditory feedback through a metronome to deliver a RAS protocol. It also provides the recording of each subject´s motor performance.
	Compass Inclinometer App [42]	Provide visual feedback during an exercise program.	The app displays three inclinometers indicating the degree of tilt of the smartphone, being a feedback input for the patients during the execution of the exercise program.
Parkinson’s Disease	Audio-biofeedback (ABF)-Gait App (CuPiD system) [41]	Recover gait through biofeedback by verbal instructions.	The app has four training targets, cadence, stride length, symmetry, and gait speed. It allows the calibration according to the subject’s gait performance and connects to the IMUs. The app provides both positive and corrective verbal feedback during the gait training.
	Freezing of Gait (FOG)-cue App (CuPiD system) [41]	Recover gait providing biofeedback by visual information.	The app allows: (1) to detect FOG, adjusted by the physiotherapist; (2) to prescribe exercise (walking in a figure of eight, making turns with and without replying to visual information, and walking through messed spaces); and (3) to provide intelligent cueing during walking and FOG episodes.
	Wellpepper App [48]	Facilitate changes in physical activity behaviour based on cognitive-behavioral content.	The app includes the videos and exercise prescription instructions (type, sets, repetitions, and auditory orders). The subjects can access their physical activity program through the app, and they also report data on pain and physical activity parameters (completed exercises and walking goals, adherence, level of difficulty) weekly. The app sends motivational notifications to encourage subjects for completing the prescribed goals, and it also allows the subjects and the physical therapist to maintain communication via text-messaging.
Multiple Sclerosis	Patient Information App (PIA) [50]	Provide educational content on physical activity and its effects on different health-related outcomes and disease progression.	The app contains diagrams and shorts videos clips of different health professionals and patients with multiple sclerosis sharing their experience with physical exercise. It provides large information about several forms of training (muscle strength, mobility, balance), their correct instructions, the risk of potential adverse effects, and the benefits on quality of life and mental health.
Neurological conditions (mixed)	Lose it!, iPro Habit Tracker and Memory: The Dairy Apps [49]	Self-management intervention for monitoring physical activity parameters and enhancing active behaviors.	The Lose it! app was used to monitor physical activity and nutrition behaviors; the iPro Habit Tracker app was used to track goals progress; and the Memories app was used to record symptoms. These apps allow subjects to visualize their progress to achieve physical activity goals by feedback functionalities. The subjects can choose preferences about information, goal setting, and self-monitoring.
	Pt Pal App[40]	Provide a home-based physical activity program.	The app provides an individualized exercise program. It includes picture or video clips of each exercise and dose exercises instructions (sets, repetitions, and number of times to perform each exercise daily). It allows subjects to carry out the exercise program in real time, guided by auditory feedback. Also, at the end of each exercise, the subjects can record the difficulty and level of pain for further review by the therapists.

Regarding the control interventions, conventional physical and occupational therapies were included, such as specific gait and balance training, home-based PA programs, and active behavior recommendations. None included study compared the mHealth group with no intervention or waitlist.

Concerning the measuring instruments, several motion analysis systems were used (GAITRite platform [47], OptoGait system [44], and ProtoKinetics platform [41]) for gait spatiotemporal analysis. Furthermore, the 10-meter Walk Test (10MWT) [40, 46] was also used for the gait speed analysis. For gait and dynamic balance analysis, the Timed Up and Go (TUG) test [40, 45–47], the Four Squared Step Test (FSST) [41, 47], the 6-minute Walk Test (6MWT) [40, 46, 48–50], the 2-minute Walk Test (2MWT) [41, 50], and the mobility subscale of the Postural Assessment Scale for Stroke (PASS) [42, 43] were used.

### Quality assessment

The overall PEDro score was 6.23 (range 3-8), implying a moderate quality of the articles included in this review. Ten studies [38, 40–45, 48–50] obtained a good methodological quality, two were acceptable [46, 47], and one was poor [39] (Online resource 3). Concerning the assessment of the RoB by domain, 7 studies had low RoB and the rest had some concerns for the allocation domain. For the second domain (bias due to deviations from intended interventions), 9 studies had low RoB while 4 studies had some concerns. In case of third (missing outcome data) and fourth (measurement of the outcomes) domains, 11 and 10 studies had low RoB, respectively. For the last domain, in the selection of the reported results, 11 studies had low RoB and only one study had some concerns (Online resource 4). Regarding the overall judgement, 5 studies had a low RoB, 5 studies had some concerns, and 3 studies had a high RoB, as shown in Online resource 4.

### Results on gait and dynamic balance

**Fig. 2:**
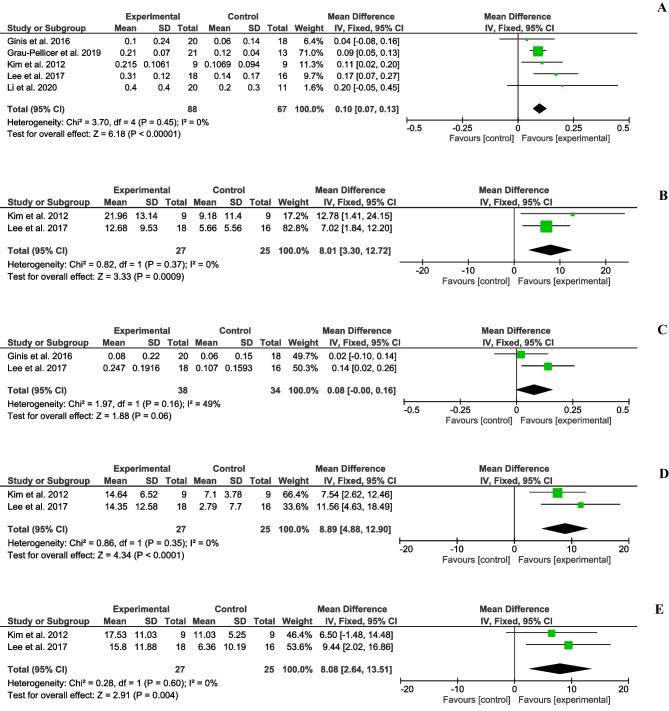
Forest plots of gait spatiotemporal parameters. (**A**) Mean difference (95% CI) of effect of mHealth vs. conventional therapy on speed; (**B**) Mean difference (95% CI) of effect of mHealth vs. conventional therapy on cadence; (**C**) Mean difference (95% CI) of effect of mHealth vs. conventional therapy on stride length; (**D**) Mean difference (95% CI) of effect of mHealth vs. conventional therapy on affected step length; (**E**) Mean difference (95% CI) of effect of mHealth vs. conventional therapy on non-affected step length; CI: Confidence interval; IV: Inverse variance; SD: Standard deviation

**Fig. 3:**
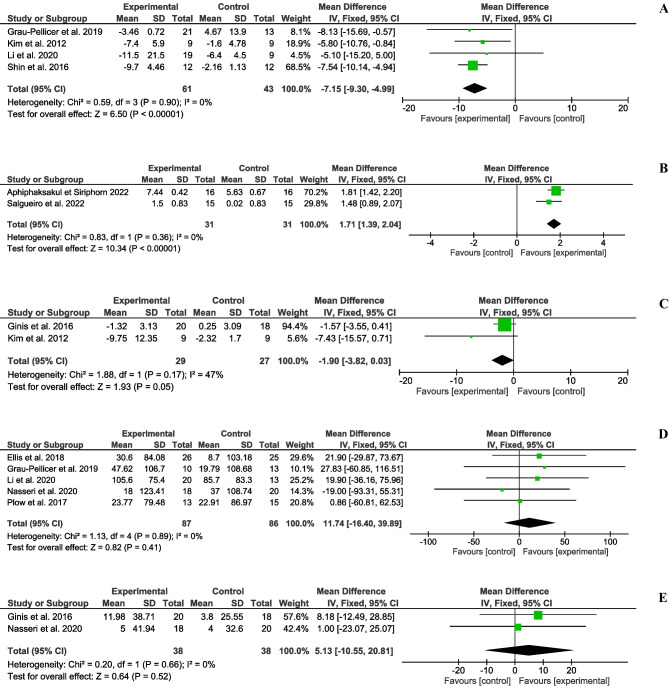
Forest plots of dynamic balance. (**A**) Mean difference (95% CI) of effect of mHealth vs. conventional therapy on TUG; (**B**) Mean difference (95% CI) of effect of mHealth vs. conventional therapy on PASS (mobility); (**C**) Mean difference (95% CI) of effect of mHealth vs. conventional therapy on FSST; (**D**) Mean difference (95% CI) of effect of mHealth vs. conventional therapy on 6MWT; (**E**) Mean difference (95% CI) of effect of mHealth vs. conventional therapy on 2MWT; CI: Confidence interval; IV: Inverse variance; SD: Standard deviation

The mean, standard deviation and sample sizes of the different subgroups and measures, along with the MD (95%CI), are shown in Figs. 2 and 3. No statistical heterogeneity was observed in seven of the ten outcomes studied. However, stride length and FSST presented moderate heterogeneity (I^2^=49% and I^2^=47%, respectively), while PASS (mobility) presented substantial heterogeneity (I^2^=75%). We found significant differences in temporal gait parameters, speed (MD=0.10;95%CI=0.07,0.13; p<0.001) (Fig. 2A) and cadence (MD=8.01;95%CI=3.30,12.72; p<0.001) (Fig. 2B). Regarding spatial gait parameters, the stride length results were no significant (MD=0.08;95%CI=-0.00,0.16; p=0.06) (Fig. 2C). Conversely, there were significant differences in affected step length outcome (MD=8.89;95%CI=4.88,12.90; p<0.001) (Fig. 2D) and non-affected step length (MD=8.08;95%CI=2.64,13.51; p=0.004) (Fig. 2E). Concerning the dynamic balance, significant differences were observed in the TUG test (MD=-7.15;95%CI=-9.30,-4.99; p<0.001) (Fig. 3A), and PASS (mobility) (MD=1.71;95%CI=1.38,2.04; p<0.001) (Fig. 3B). The FSST (MD=-1.90;95%CI=-3.82,0.03; p>0.05) (Fig. 3C), 6MWT (MD=11.74;95%CI=-16.40,39.89; p=0.41) (Fig. 3D), and 2MWT (MD=5.13;95%CI=-10.55,20.81; p=0.52) (Fig. 3E) results were no significant. Therefore, the results of the present study suggest that the use of mHealth systems was slightly more effective than the conventional rehabilitation of training and enhancing gait and balance outcomes according to motion analysis systems, the TUG test, and the PASS (mobility).

## Discussion

The present systematic review and meta-analysis were performed to analyze the impact of the use of mHealth systems on gait and dynamic balance outcomes in subjects with neurological disorders. To our knowledge, this is the first systematic review and meta-analysis analyzing the use of mHealth systems for improving gait and balance in neurorehabilitation, providing the first evidence-based findings supporting its clinical application. As a result, 13 studies were reviewed and 11 were examined in this meta-analysis. Therefore, and according to our results, there was some evidence of the use of mHealth systems which can impact positively on gait and balance outcomes in subjects with neurological disorders.

There are some reviews [13, 29–31] of both literature and apps markets conducted to analyze the commercial mobile apps in the neurorehabilitation field. They stated that despite the wide variety of available apps with potential use and design for subjects with neurological disorders, the scientific evidence from RCT is also limited to generalize their efficacy and merge their recommendation. In addition, they recommended knowing the characteristics and therapeutic needs of this population to develop well-founded and feasibility apps. Accordingly, a recent review [51] also recommended the need to design new and mixed teleneurorehabilitation protocols, which will be focused on measuring quantitative and qualitative measures simultaneously. Another widespread conclusion in mHealth research is the content validation, for which there are several evaluation tools [52].

Gait recovery is the key goal in neurorehabilitation [53], and new modalities of intervention are emerging. In that way, one of these modalities is the use of the mHealth systems for improving spatiotemporal gait parameters, and those parameters are reliable measurements to quantify gait recovery [54]. There are different systems for recording, assessing, and monitoring motor impairments in daily life settings for the population with neurological disorders. In our meta-analysis, the findings on walking speed, cadence, and affected and non-affected step length showed significant improvements, so it can be considered a promising and effective training method to improve those parameters. Nevertheless, the results should be taken with caution because of the differences in assessment systems and tests used, studied population and the lightly studies included.

Gait impairments are closely related to decreased motor trunk control and postural balance. The involvement of similar brain centers and neural pathways could explain this relationship for integrating neuromuscular and sensory signals [55]. Therefore, neurorehabilitation should aim to improve both functions to facilitate daily life activities recovery for independent and social participation [56], as well as to decrease the risk of falls in population with neurological disorders [41]. In this sense, our results showed controversial findings depending on the measuring instrument. It is important to highlight that the study using the CSMi apps [45], is the only intervention designed for balance training, measuring the dynamic balance with the TUG test, but not with FSST and 6MWT. It might influence our results, getting significant results for the TUG test, but not for FSST, 6MWT and 2MWT.

The improvements in gait and balance using mHealth could be enhanced by several strategies, such as auditory feedback, visual feedback, or incentive-based approaches. Concerning the ZyMi Metronome App [47] and GotRhythm App [39], they provide gait training through rhythmic auditory stimulation for exciting motor neurons in the brainstem and the spinal level, promoting motor learning and changes in neuroplasticity through audition [57]. In that way, there is wide evidence of the rhythmic auditory stimulation effects in recovering spatiotemporal gait parameters and dynamic balance in subjects with neurological disorders [58–60]. Furthermore, ABF-gait App [41] provided auditory feedback using verbal corrections and positively reinforce during gait performance. Moreover, there are smartphone apps used in the included studies which provide visual feedback, such as Virtual Active App [44], FOG-cue App [41], CSMi Apps [45], Compass Inclinometer App [42], or Farmalarm App [43], during the intervention to guide motor performance. According to Noh et al. [56], it may be more effective than conventional training for gait and balance recovery. Finally, there are incentive-based mHealth apps, such as Wellpepper App [48], Fitlab Training App [46], iPro Habit Tracker App [49], Pt Pal App [40], Patient App [38] or PIA [50], which provide home-based PA programs, PA information and recommendations with activity goal attainments and rewards, increasing the subjects’s motivation and the adherence to PA [46].

Concerning the baseline characteristics of the participants, eight studies [38, 42–47] included subjects with stroke, in subacute or chronic phases. Two studies [41, 48] involved subjects with Parkinson’s disease, other two [40, 49] included a sample of subjects with several neurological disorders, and one study included subjects suffering multiple sclerosis [50]. Therefore, the functional characteristics of the included participants were heterogeneous. In addition, the sample size was relatively limited in most included studies with only 20-30 participants, making it difficult to establish adequate and solid conclusions. It is important to consider that these types of studies are usually performed in clinical or home settings, without controlling the environmental influence and involving patients receiving treatment in an institution. Therefore, it is necessary to perform multi-centered studies with no convenience samples, so it may generate selection bias [61].

Aiming to complete our insight about the analyzed topic, the minimum clinically important differences (MCID) were considered evaluating the clinical relevance of our results. According to the speed gait parameter, it was estimated that the MCID in subjects with stroke is 0.16 m/s [62] for subacute phases and an overall value of 0.13 m/s [63] was determined. For Parkinson’s disease, it was established as the range of clinically important difference an enhanced speed gait from 0.05 to 0.22 m/s, considering 0.14 m/s as moderate change [64]. In any case, despite the statically significant difference in gait speed between studied groups, it cannot be considered a clinically relevant change. Regarding dynamic balance, the MCID of the TUG test is stated as -3.8 to 2.6 seconds for subjects with stroke [65]. In this instance, our results of the TUG test reached that clinical relevance. Although the overall results of our meta-analyses were significant for cadence, step length and PASS (mobility) in subjects with neurological diseases, the information about the MCID was not found. Therefore, we recommend the scientific community provide future studies results in this way.

Finally, regarding the role of the neurorehabilitation professionals, they have an active role on guiding patients on the effective use of mHealth tools and ensuring that the interventions are safe and well-conducted [66]. By doing so, they can help patients managing their conditions more effectively and improving their overall health outcomes. They also assist patients, families, and caregivers to have clear criteria and indicators to use the optimal mHealth tools for their specific requirements [30]. Definitively, professionals need to know the validation data and scientific evidence of each app, as well as their approval by the corresponding control agencies [67], in order to individualize the clinical recommendations to the needs of the patients with neurological disorders.

### Study limitations

Although this review and meta-analysis provide an evidence-based overview of the use of mHealth in subjects with neurological disorders, some limitations should be remarked. Our results should be taken with caution because of the heterogeneity of neurological disorders and the variety of assessment tools used. Likewise, several intervention protocols were used in experimental and control groups. These facts could influence the comprehension and interpretation of findings. Thus, protocols standardization is needed for future studies. Another common limitation, along with the studies included, was the lack of assessor blinding for group allocation, resulting in a high risk of bias in this domain. As well as stated previously, the sample size involved in the studies was limited, making the generalization of the obtained results difficult. Finally, it is necessary to perform trials with long-term follow-up to assess whether the mHealth effects are maintained in neurorehabilitation.

Nevertheless, despite of those limitations, this systematic review and meta-analysis provides the first evidence-based findings about the use of mHealth systems for gait and balance recovery in neurorehabilitation. Therefore, this manuscript could be used as the base of future clinical studies and discover the necessity of further research that will allow the stratified analysis of the results.

## Conclusions

In conclusion, our study showed some evidence that mHealth systems can be a useful tool for subjects with neurological disorders to improve gait, and controversial results were found on dynamic balance recovery. In that way, the use of smartphones and tablets for performing clinical or home-based rehabilitation is still in its first stages, and the quality of evidence is not enough to merge the effectiveness and strongly recommend the use of these systems in neurorehabilitation. Thus, it is needed to conduct clinical trials with higher methodological quality, involving a larger sample size, homogenous studied groups, standardized protocols, and outcome measures to recommend mHealth as an alternative or reliable complement to clinical neurorehabilitation.

### Supplementary Information

Below is the link to the electronic supplementary material.

Supplementary file1 (PDF 79 KB)

Supplementary file2 (PDF 75 KB)

Supplementary file3 (PDF 54.1 KB)

Supplementary file4 (PDF 357 KB)
