# Supplementary material for: mHealth Impact on Gait and Dynamic Balance Outcomes in Neurorehabilitation: Systematic Review and Meta-analysis
Source: J Med Syst. 2023 Jul 18;47(1):75. doi: 10.1007/s10916-023-01963-y (PMC10354142; doi:10.1007/s10916-023-01963-y)
Supplement: Supplementary file 2 — Supplementary file2 (PDF 75 KB) [file 10916_2023_1963_MOESM2_ESM.pdf]

Online resource 2. Complete search strategy	
**#1**	Search (“neurolog*” [MesH] OR neurologia OR neurologic OR neurological OR neurologically OR neurologist OR neurologists OR neurology)
**#2**	Search (“smartphone” OR “mobile” OR “mhealth” OR “m-health”)
**#3**	Search (“randomized controlled trial” OR “controlled trial” OR “randomized clinical trial” OR “randomised controlled trial” OR “randomised clinical trial”)
**#5**	Search (#1 AND #2 AND #3)
