# Supplementary material for: mHealth Impact on Gait and Dynamic Balance Outcomes in Neurorehabilitation: Systematic Review and Meta-analysis
Source: J Med Syst. 2023 Jul 18;47(1):75. doi: 10.1007/s10916-023-01963-y (PMC10354142; doi:10.1007/s10916-023-01963-y)
Supplement: Supplementary file 3 — Supplementary file3 (PDF 54.1 KB) [file 10916_2023_1963_MOESM3_ESM.pdf]

Online resource 3. PEDro scale score for clinical trials included in the review.

Studies	Score	Methodology quality	1	2	3	4	5	6	7	8	9	10	11
Ginis et al., 2015	6	Good	-	1	0	1	0	0	0	1	1	1	1
Kim et al., 2012	5	Acceptable	-	1	0	1	0	0	0	1	0	1	1
Ellis et al., 2018	7	Good	-	1	1	1	0	0	1	1	0	1	1
Plow & Golding., 2017	6	Good	-	1	1	0	0	0	1	1	0	1	1
Grau-Pellicer et al., 2019	4	Acceptable	-	1	0	1	0	0	0	0	0	1	1
Lee et al., 2017	6	Good	-	1	0	1	0	0	1	1	0	1	1
Shin & Song., 2016	6	Good	-	1	0	0	1	0	1	1	0	1	1
Nasseri et al.,2020	8	Good	-	1	1	1	0	0	1	1	1	1	1
Asano et al., 2021	7	Good	-	1	1	1	0	0	1	0	1	1	1
Li et al., 2020	7	Good	-	1	0	1	0	0	1	1	1	1	1
Hankinson et al., 2022	3	Poor	-	1	1	0	0	0	1	0	0	0	0
Salgueiro et al., 2022	8	Good	-	1	1	1	0	0	1	1	1	1	1
Aphiphaksakul et Siriphorn, 2022	8	Good	-	1	1	1	0	0	1	1	1	1	1
Ranged 0-10. Item 1 is related to external validity and not used in the method score.													
