# Supplementary material for: mHealth Impact on Gait and Dynamic Balance Outcomes in Neurorehabilitation: Systematic Review and Meta-analysis
Source: J Med Syst. 2023 Jul 18;47(1):75. doi: 10.1007/s10916-023-01963-y (PMC10354142; doi:10.1007/s10916-023-01963-y)
Supplement: Supplementary file 4 — Supplementary file4 (PDF 357 KB) [file 10916_2023_1963_MOESM4_ESM.pdf]

Online Resource 4. Risk of bias of studies included in the systematic review.

Study	D1	D2	D3	D4	D5	Overall
	Risk of bias domains					
Asano et al., 2021	+	+	-	+	+	-
Ellis et al., 2018	+	+	+	+	+	+
Ginis et al., 2016	+	+	+	-	+	-
Grau-Pellicer et al., 2019	-	-	+	-	+	X
Kim et al., 2012	-	-	+	-	+	X
Lee et al., 2017	+	+	+	+	+	+
Li et al., 2020	-	+	+	+	+	-
Nasseri et al., 2020	+	+	+	+	+	+
Plow & Golding., 2017	-	+	+	+	+	-
Shin & Song., 2016	-	-	+	+	+	-
Hankinsonson et al., 2022	-	-	X	+	-	X
Salgueiro et al., 2022	+	+	+	+	+	+
Aphiphaksakul et Siriphorn, 2022	+	+	+	+	+	+

Domains:
D1: Bias arising from the randomization process.
D2: Bias due to deviations from intended intervention.
D3: Bias due to missing outcome data.
D4: Bias in measurement of the outcome.
D5: Bias in selection of the reported result.

Judgement
X High
- Some concerns
+ Low
